# Genetic Mechanisms Involved in Microbial Stress Responses

**DOI:** 10.3390/genes15101265

**Published:** 2024-09-27

**Authors:** Jose M. Requena

**Affiliations:** Centro de Biología Molecular Severo Ochoa (CSIC-UAM), Departamento de Biología Molecular, Instituto Universitario de Biología Molecular (IUBM), Universidad Autónoma de Madrid, 28049 Madrid, Spain; jmrequena@cbm.csic.es

The ability of living beings to deal with abrupt environmental changes is paramount for survival, and organisms have evolved a large variety of molecular mechanisms (known globally as stress responses) to buffer the harmful effects of stressors on cellular homeostasis [1]. Cells respond to stress stimuli through coordinated changes in gene expression; often translational and post-translational regulation are used under these circumstances because they allow for immediate and selective changes in protein levels and activity. The seven articles comprising this Special Issue give an overall view of the variety and complexity of the mechanisms and molecules involved in the stress responses analyzed in different microorganisms (either prokaryotes or eukaryotes). Figure 1 graphically summarizes the main contents studied and described in this collection of articles. Also, from a methodological point of view, these articles contribute detailed experimental procedures and bioinformatic tools that may prove useful for conducting similar studies on different organisms.

During optimization of productive processes in industrial-scale bioreactors, many often unforeseen performance losses occur when transferring a process from the homogeneous laboratory environment to the industrial scale. In a study by Minden and colleagues (University of Stuttgart, Germany), the effect that transient glucose deprivation (2 min glucose depletion) exerts on the whole transcriptome was analyzed in a well-adapted *Saccharomyces cerevisiae* strain for ethanol production [2]. After restoring normal glucose levels, samples were obtained at several time intervals and processed for next-generation sequencing of RNA (RNA-seq). Remarkably, this brief glucose shortage (a fluctuation easily produced in bioreactors during feeding and mixing procedures) led to a differential expression of 1053 genes. After functional grouping of those genes, the authors suggested that stress response–growth trade-off processes were induced, being primarily balanced via the upstream effector target of rapamycin 1 (TORC1), protein kinase A (PKA), mitogen-activated kinase (MAPK) cascade and the glucose de-repression program [3]. In sum, the defensive transcriptional program elicited by extremely short glucose deprivation is aimed at inducing a global stress response and growth repression. Hence, the primary objective of the yeasts is to save resources by ramping down growth capacities to invest in survival strategies. However, this poses several inconveniencies, such as decreased product yields and accumulation of undesired by-products during industrial production processes. A final message from this study is that pre-adaptation of strains to this insult (glucose deprivation) may be a strategy for optimizing the industrial-scale production of bioreactors.

In the article by Asante and colleagues (University of Cape Coast, South Africa), the capacity of biofilm formation was investigated genotypically in a large collection of antibiotic-resistant clinical isolates of *Staphylococcus epidermidis* coagulase-negative strains [4]. Coagulase-negative staphylococci (CoNS) are fearsome pathogens responsible for causing urinary tract infections, bloodstream infections, and endocarditis in millions of people around the world [5]. The authors have elaborated an excellent introduction on biofilms (communities of microorganisms, mainly bacteria) and present them as structures aimed at resisting environmental stress conditions, including the presence of antimicrobial agents. Also, in infected persons, many pathogens promote biofilm formation to evade host defense mechanisms. Moreover, staphylococcal virulence factors and the molecular processes involved in biofilm formation have been described clearly and concisely. For the research work described in this article, the authors phenotypically characterized a collection of CoNS blood culture isolates using well-established procedures. Subsequently, a sub-sample of 18 methicillin-resistant CoNS isolates was selected for whole-genome sequencing (WGS); bioinformatics analyses were conducted to detect genes encoding adherence factors and proteins involved in biofilm formation. As a result, the authors provided a detailed table with the biofilm-forming factors present in each of the 18 isolates that was analyzed. This knowledge is essential to understand the mechanisms of biofilm formation and will be useful in developing compounds that prevent or break down biofilms once established.

TOR (Target of Rapamycin) is a serine/threonine kinase that controls cell growth in eukaryotes in response to nutrients and energy availability, modulating general processes such as transcription, ribosome biogenesis, and translation [6]. Under stress conditions, the TOR signaling pathway is inhibited. The experimental objective, described in the article by Santiago and colleagues (Universidad de Jaén, Spain), was to unravel the participation of protein Bud27 in the mechanisms governed by the TOR signaling pathway [7]. The orthologue to *S. cerevisiae* Bud27 in humans is URI, a molecular chaperone of the TOR complex that is involved in coordinating gene expression according to nutrient availability. Moreover, Bud27 is also present in the three RNA polymerase molecular complexes (I, II, and III). The experimental approach followed by these authors was to transfect a temperature-sensitive bud27Δ mutant line with a genomic DNA library looking for clones able to grow at 37 °C. As expected, six out of the 71 selected clones had incorporated the wild-type (WT) BUD27 gene. In addition, nine and 26 clones contained plasmid coding for proteins RPB5 or RPB6, respectively. These findings agree with a previous report showing that overexpression of these proteins overcame the temperature sensitivity of the bud27Δ mutant [8]. Other clones, in which the temperature sensitivity of the mutant was overcome, contained plasmids coding for the Rpc17 subunit of RNA pol III, the protein Sun4, several large ribosomal proteins (Rpl40a, Rpl8a, Rpl34a, and Rpl33b), Smy2, Frs2, Msd1, Pab1, Flc3, Spo20, Ssp2, Gcr1, Atg39, Atg36, and Pex8. Additional experiments with the bud27Δ mutant indicated that the absence of Bud27 leads to cellular stress, which affects cell wall integrity (CWI) and slows autophagy induction.

Apart from their essential role in digestion and nutrient absorption in the gut, bile salts exert antimicrobial effects because of their capacity to dissolve membrane lipids and cause the denaturation of proteins. However, the pathogen *Salmonella enterica* shows marked resistance to bile salts, being able to establish and survive in the gall bladder during chronic infections. In the article by Lyu and colleagues (University of Maryland, College Park, MD, USA), it is reported that resistance to sodium cholate positively correlates with an increased ribosomal fidelity [9]. The rate of amino acid misincorporation during translation increases under external stresses such as heat, and stressed organisms respond by blocking ribosomal protein synthesis [10]. For this work, the authors designed a dual-fluorescence reporter readthrough assay to determine the error rate during translation. Another valuable resource these authors generate is a large collection of *Salmonella* mutant lines. The main conclusion raised in this study is that increased ribosomal fidelity provides a growth advantage for *Salmonella* under bile salt stress. The authors hypothesize that cells featuring high-fidelity translation invest less ATP in eliminating incorrect proteins and that the resulting higher ATP levels, in turn, benefit growth under stress conditions.

*Giardia intestinalis* is an intestinal protozoan parasite causing giardiasis, a severe diarrheal disease affecting humans and other mammals in many countries. During transmission, to resist environmental stressful conditions, the parasite has evolved an encystation process that leads to the formation of infectious cysts [11]. In the article by Rojas-López and colleagues (Uppsala University, Uppsala, Sweden), the authors conducted a detailed transcriptomic analysis of the gene expression changes occurring along the time course of *G. intestinalis* encystation [12]. For this study, a high-bile encystation protocol was used to differentiate trophozoites into cyst form, and the authors studied gene expression changes every 3.5 h of the encystation process using massive RNA sequencing (RNA-seq). A gradual alteration in gene expression was noted; after 3.5 h encystation, there were only 20 differentially expressed genes (DEGs), whereas the amount of DEGs reached 3409 in the cyst stage (after 3 days of encystation induction). Not surprisingly, in the cysts, gene coding for variable surface proteins (VSPs), which are involved in antigenic variation for immune evasion, were among the main differentially expressed elements. The authors also noted an early activation of the encystation-associated transcription factor Myb2 (GL50803_8722) and a stepwise upregulation of other transcription factors, including EF2/DP (GL50803_23576) at 10.5 h post-induction (pi), ARID2 (GL50803_8102) at 14 h pi, GARP-like 2 (GL50803_88945) at 21 h pi, and GARP-like 3 protein (GL50803_9154) at 28 h pi. For researchers working in this research field or interested in the molecular aspects occurring during the encystation process (common to many parasites), this article provides a high-resolution gene expression map that can guide future studies aimed at deciphering this important cellular differentiation process, which is critical for the transmission of this deadly parasite.

Lactic acid bacteria (LAB) are widely used in the food industry because of their beneficial contribution to the elaboration of many alimentary products; however, in products like juices, beverages, and beer, microorganisms have to be eliminated to avoid spoilage or other undesired effects. High hydrostatic pressure (HHP) is often used as a preservation method to reduce the number of microbial counts in beverage products. Nevertheless, under HHP conditions, some LAB species induce stress responses that allow them to survive those drastic environmental insults. The aim of the study reported by Bucka-Kolendo and colleagues (Prof. Wacław Dabrowski Institute of Agricultural and Food Biotechnology-State Research Institute, Warsaw, Poland) was to analyze the effect of HHP (300 MPa/5 min) on the expression levels of three stress-related genes (dnaK, hrcA, and ctsR) in 15 lactobacilli strains used in food production [13]. DnaK is the name used in bacteria to refer to the universally ubiquitous chaperone HSP70, a key player in stress responses. HrcA and CtsR are positive and negative, respectively, transcriptional regulators of the heat shock GroES-GroEL and HrcA-DnaK-GrpE-DnaJ operons in *Lactobacillus* [14]. After HHP treatment, the authors determined the viability of each strain by counting colony-forming units while, in parallel, extracting RNA for analyzing the expression levels of the above-mentioned genes by real-time PCR (RT-qPCR). Substantial differences in cell viability were observed among the analyzed strains, and the RT-qPCR data indicate that gene expression levels of dnaK, ctsR, and hrcA can be useful markers to monitor the LAB cellular response to HHP. These molecular parameters can help in optimizing the conditions used in industrial food processes to achieve products of better quality. 

To quickly respond to harsh environmental conditions (nutrient deprivation, hypoxia, oxidative stress, and heat shock, among others), organisms have evolved families of molecular chaperones designed to operatively maintain protein interaction networks until specific responses are mounted to alleviate cellular alterations. Many of the molecular chaperones were first uncovered because of their increased induction after heat shock treatments and, therefore, are named as heat shock proteins (HSPs). Nevertheless, HSPs also serve as central integrators of cellular homeostasis during normal cell growth and differentiation. In the article by Solana and colleagues (Centro de Biología Molecular Severo Ochoa, Spain), *Leishmania infantum* genome-wide mining for gene coding for proteins of the HSP40 family was conducted [15]. These proteins, also known as DnaJ-domain-containing proteins (JDPs) or J-proteins, are co-chaperones for members of the HSP70 family, which is considered to be the leading player in cellular protein homeostasis [16]. *Leishmania* parasites are the causative agent of leishmaniasis, an insect-born disease affecting humans and other mammals. These authors identified 72 different HSP40 proteins, categorized into four groups according to structural features. The number of distinct HSP40s existing in *Leishmania* is greater than those found in humans and other model organisms; to date, only the HSP40 family in pepper, with 76 members, outnumbers the *Leishmania* one. A better knowledge of these proteins and their functional relevance will bring opportunities for drug discovery and improvement of current treatments against leishmaniasis.

In sum, I hope readers find these contributions to be scientifically sound and nobody ignores them because of the journal’s name.

## Figures and Tables

**Figure 1:**
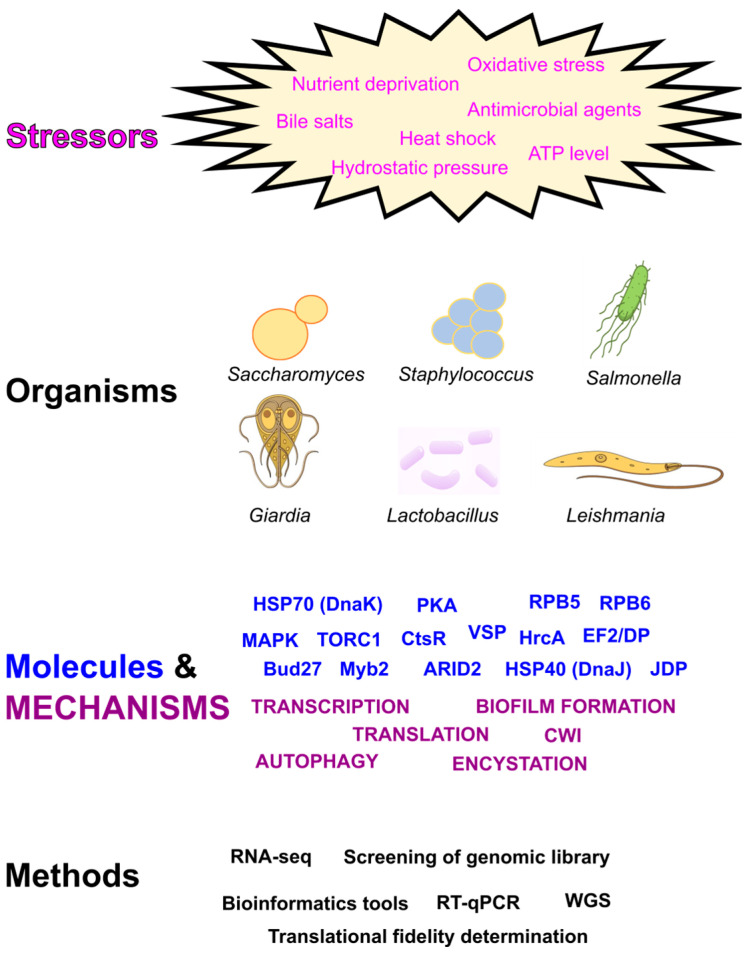
Schematic overview of the contents included in the articles comprising this Special Issue. Abbreviations: CWI, cell wall integrity; JDP, DnaJ-domain-containing protein; MAPK, mitogen-activated kinase; PKA, protein kinase A; RNA-seq, next-generation sequencing of RNA; TORC1 (target of rapamycin 1); VSP, variable surface protein; WGS, whole-genome sequencing.
